# Genetic polymorphism in selenoprotein P modifies the response to selenium-rich foods on blood levels of selenium and selenoprotein P in a randomized dietary intervention study in Danes

**DOI:** 10.1186/s12263-018-0608-4

**Published:** 2018-07-13

**Authors:** Tine Iskov Kopp, Malene Outzen, Anja Olsen, Ulla Vogel, Gitte Ravn-Haren

**Affiliations:** 1 National Food Institute, Technical University of Denmark, Kemitorvet, Building 202, 2800 Kgs Lyngby, Denmark; 2 Danish Cancer Society Research Center, Strandboulevarden 49, 2100 Copenhagen Ø, Denmark; 3 The Danish Multiple Sclerosis Registry, Copenhagen University Hospital, Rigshospitalet, Blegdamsvej 9, 2100 Copenhagen Ø, Denmark; 4 National Research Centre for the Working Environment, Lersø Parkallé 105, 2100 Copenhagen Ø, Denmark; 5 The Danish Multiple Sclerosis Center, Department of Neurology, The Danish Multiple Sclerosis Registry, Section 7801, Rigshospitalet, Blegdamsvej 9, 2100 Copenhagen Ø, Denmark

**Keywords:** Selenium, Selenoprotein P, Glutathione peroxidase, Dietary intervention, Randomized, Gene-diet interaction, *SELENOP*, *GPX1*, *GPX4*, Single nucleotide polymorphisms

## Abstract

**Background:**

Selenium is an essential trace element and is suggested to play a role in the etiology of a number of chronic diseases. Genetic variation in genes encoding selenoproteins, such as selenoprotein P and the glutathione peroxidases, may affect selenium status and, thus, individual susceptibility to some chronic diseases. In the present study, we aimed to (1) investigate the effect of mussel and fish intake on glutathione peroxidase enzyme activity and (2) examine whether single nucleotide polymorphisms in the *GPX1*, *GPX4*, and *SELENOP* genes modify the effect of mussel and fish intake for 26 weeks on whole blood selenium, plasma selenoprotein P concentrations, and erythrocyte GPX enzyme activity in a randomized intervention trial in Denmark.

**Results:**

CC homozygotes of the *SELENOP*/rs3877899 polymorphism who consumed 1000 g fish and mussels per week for 26 consecutive weeks had higher levels of both selenoprotein P (difference between means − 4.68 ng/mL (95% CI − 8.49, − 0.871)) and whole blood selenium (difference between means − 5.76 (95% CI − 12.5, 1.01)) compared to fish and mussel consuming T-allele carriers although the effect in whole blood selenium concentration was not statistically significant.

**Conclusions:**

Our study indicates that genetically determined variation in *SELENOP* leads to different responses in expression of selenoproteins following consumption of selenium-rich foods. This study also emphasizes the importance of taking individual aspects such as genotypes into consideration when assessing risk in public health recommendations.

**Electronic supplementary material:**

The online version of this article (10.1186/s12263-018-0608-4) contains supplementary material, which is available to authorized users.

## Background

Selenium is an essential trace element and is suggested to play a role in the etiology of a number of chronic diseases [9, 37]. Several factors can affect selenium status; these include exposure to selenium through diet or dietary supplements [32, 33, 35] and lifestyle factors such as body mass index (BMI) and smoking [6]. Furthermore, genetic variation has been proposed to influence selenium status of the individual [14].

Most of the biological functions of selenium are carried out by selenoproteins [30]. Among the best characterized selenoproteins with essential functions are selenoprotein P and the glutathione peroxidases (GPX), including GPX1 and GPX4 [30]. Besides functioning as storage and transport protein for selenium, some studies suggest that selenoprotein P is involved in the antioxidant defense which also comprises the glutathione peroxidases [40]. These antioxidant selenoenzymes detoxify a range of hydroperoxides, including lipid and phospholipid hydroperoxides, thereby ensuring a reducing environment and protecting cell components such as proteins, lipids, and DNA from oxidative damage [36]. Nutritional deficiency in selenium results in decreased selenoprotein concentrations and a compromised enzymatic antioxidant defense. Selenium supplementation is known to affect selenoprotein expression in a hierarchical manner. GPX4 ranks higher in the hierarchy of selenoproteins compared to GPX1 which is much more responsive to both selenium depletion and repletion [2, 4]. Humans differ in their ability to metabolize selenium and respond differently to selenium supplementation. These differences are likely to be due to genetic variation in selenoprotein genes [6, 16]. Specifically, functional single nucleotide polymorphisms (SNPs) in *GPX1* (encoding GPX1), *GPX4* (encoding GPX4), and *SELENOP* (encoding SELENOP) have been shown to affect blood selenium or selenoprotein levels in response to supplementation [19, 20, 23]. The *GPX1*/rs1050450 polymorphism is a Pro to Leu substitution at position 197 [10] which results in reduced enzyme activity [16, 34] and higher DNA damage levels [25]. *GPX1*/rs1050450 has also been associated with risk of several diseases, including lung [31, 39], breast [8, 21, 34], and prostate cancer [3, 43]; peripheral neuropathy [45]; coronary heart diseases [26, 50]; septic shock [18]; and mortality [42], and correlation between *GPX1* activity and serum selenium concentration has been reported for this polymorphism [16]. *GPX4*/rs713041 affects protein binding to the 3′-untranslated region close to a selenocysteine insertion sequence required for selenoprotein synthesis [20, 48] and has been associated with decreased GPX enzyme activity [21], risk of colorectal cancer [43], and mortality [47]. *SELENOP*/rs3877899 causes an Ala to Thr amino acid substitution at position 234, and *SELENOP*/rs7579 is located in the 3′-untranslated region of *SELENOP* mRNA, where it causes a G to A base change [19]. Both *SELENOP* polymorphisms lead to alterations in selenium metabolism by changing the proportion of the 60-kDa isoform of selenoprotein P [23]. They have been shown to affect selenium and selenoprotein P blood concentrations after supplementation with selenium [19] and have also been associated with risk of various diseases, including breast [21], prostate [7, 13, 43], and colorectal cancer [22, 23] and aortic aneurisms [44]*.*Taken together, these results point to a regulatory role of these polymorphisms in the expression of the encoded selenoproteins. However, it is not clear whether this is also the case at selenium intakes that are dietary feasible.

In a previous randomized dietary intervention with fish and mussels in middle-aged Danes, we found increased whole blood selenium and plasma selenoprotein P concentrations among healthy participants after 26 weeks’ intervention [28]. We hypothesized that *SELENOP* gene variations modify the effect of increased dietary selenium intake on markers of selenium status. Thus, in the present paper, we aimed to (1) investigate the effect of mussel and fish intake on GPX enzyme activity and (2) examine whether SNPs in the *GPX1*, *GPX4*, and *SELENOP* genes modify the effect of the intervention with mussel and fish on whole blood selenium, plasma selenoprotein P concentrations, and erythrocyte GPX enzyme activity in the same study population of healthy middle-aged Danish men and women.

## Methods

### Study population

This study is based on data from a randomized dietary intervention study with the primary aim of studying the influence of increased intake of fish and mussels on blood selenium levels. The study design and methods are described in details elsewhere [28].

The study took place in the northern part of Jutland, Denmark, from September 2010 to March 2011 as a 26-week randomized dietary intervention study with two parallel groups including healthy middle-aged participants. Eligibility for randomization was determined by potential participants completing a questionnaire 3 months before baseline measurements on lifestyle, diet, and health status.

The goal was to recruit a total of 100 men and women aged 50–74 years with a BMI of 18.5–28 kg/m^2^ based on a power calculation where a minimum detectable difference of 10 ng/mL (SD = 10) or 30 ng/mL (SD = 10) in selenium concentration between groups with a statistical power of 87 or 99%, respectively, was allowed [29]. The exclusion criteria included current smoking, intake of dietary supplements containing selenium 3 months before baseline measurements, frequent intake of fish and shellfish (> 300 g/week), excessive intake of alcohol (according to the official Danish guidelines at study recruitment: women > 14 units of alcohol/week, men > 21 units of alcohol/week), strenuous exercise (> 10 h/week), severe chronic disease, and frequent use of specified medication (including diabetic medicine, anticoagulant medicine, and medication for heart disease), or a cancer diagnosis within the past 5 years. Study participants were requested to inform the investigators of any changes regarding disease or medication occurring during the study. Study participants were recruited through local media, including newspaper advertisements. Of the 102 men and women enrolled in the study, 83 completed the intervention. Flow chart of the participants is illustrated in Additional file 1 as previously published [28].

### Dietary intervention

Participants in the intervention group were provided with 1000 g raw fish and raw or processed mussels (portion size of 200 g; five portions/week) once a week for 26 weeks. This amount corresponds to an intake of approximately 50.3 μg selenium/day (based on data from the Danish Food Composition Databank) [41]. The participants received four or five different types of fish per week. Diversity in the type of fish provided was prioritized to ensure variation and thereby optimize compliance. The participants were allowed to consume other meals containing fish or shellfish besides the experimental foods. The intervention diet has been described in detail elsewhere [28].

To monitor their compliance, the participants were provided with a self-monitoring record and kitchen scales to weigh the amount of received fish and mussels (prepared or raw) not consumed during the study period. At study initiation, participants were instructed on how to complete the self-monitoring record. Compliance was calculated as total received amount—not ingested amount after preparation)/by total received amount; the median of ingested proportion was 99% of the received fish and mussels [28].

Participants in the control group received no intervention and were advised to maintain their habitual diets.

### Data collection

Non-fasting blood samples were collected three times (weeks 0, 13, and 26) from each participant during the study. Blood samples were drawn in K2-EDTA-coated blood drawing tubes as whole blood samples or blood samples that were separated into plasma, erythrocytes, and buffy coat by centrifugation. All samples were stored at − 80 °C until analysis.

### Biochemical analyses

Whole blood selenium analyses were conducted using an ELAN 6100 DRC inductively coupled plasma-mass spectrometer (ICP-MS) in accordance with a method described in detail in [15]. The concentration of selenoprotein P in plasma was determined using its selective retention by heparin-affinity high-performance liquid chromatography (HPLC) and online detection by ICP-DRC-MS of selenium eluting from the HPLC column. In addition to determining selenoprotein P, the concentration of total selenium in plasma was also quantified by isotope dilution on the basis of the area under the complete chromatogram. However, even though the results seemed accurate (that is, the values obtained from plasma selenium did not deviate from certified values from reference material), the method used to determine plasma selenium in this way was less precise than that used to determine selenium in whole blood. Therefore, the baseline plasma selenium concentration was used only for comparison with other studies measuring plasma selenium in healthy populations. The analysis methods have been described in details previously [28].

GPX enzyme activities were spectrophotometrically assayed in erythrocyte lysates on a Pentra 400 (Horiba ABX, Montpellier, France), using t-butylhydroperoxide as substrate according to the method described by Wheeler et al. [49]. GPX activities were related to the amount of hemoglobin (Hb) in the lysates. Hb content was determined using a commercially available kit (Randox Ardmore, UK, cat. no HG 980). Samples from each individual were run in the same batch in random order to decrease variation, and a control sample was included for every 15th sample. Intra- and inter-day variations were < 5 and < 10%, respectively.

### SNP selection and genotyping

The following polymorphisms were selected based on their known functionality and association with disease: *GPX1*/rs1050450, *GPX4*/rs713041, *SELENOP*/rs3877899, and *SELENOP*/rs7579.

DNA from the participants was extracted from frozen lymphocytes as described [24]. Genotypes were determined using RT-PCR and allelic discrimination on ABI 7900HT instruments (Applied Biosystems, Nærum, Denmark). Generally, 40–200 ng/μl DNA was obtained and 10 ng of DNA was genotyped in 5 μl containing 50% 2 × Mastermix (Applied Biosystems, Nærum, Denmark), 100 nM probes, and 900 nM primers.

*GPX1*/rs1050450: primers: F: 5′-TGT GCC CCC TAC GCA GGT ACA-3′, R: 5′-CCC CCG AGA CAG CAG CA-3′ (TAGCopenhagen, Copenhagen, Denmark), T-allele: 5′-FAM-CTG TCT CAA GGG C**T**C AGC TGT-MGB-3′, C-allele: 5′-VIC-CTG TCT CAA GGG C**C**C AGC TGT-MGB-3′ (Applied Biosystems, Nærum, Denmark).

*GPX4*/rs713041: primers: F: 5′-CCC ACT ATT TCT AGC TCC ACA AGT G-3′, R: 5′-GTC ATG AGT GCC GGT GGA A-3′ (TAGCopenhagen, Copenhagen, Denmark), T-allele: 5′-FAM-ACG CCC T**T**G GAG C-MGB-3′, C-allele: 5′-VIC-ACG CCC T**C**G GAG C-MGB-3′ (Applied Biosystems, Nærum, Denmark).

*SELENOP*/rs3877899 was determined using the TaqMan® Pre-designed SNP genotyping; assay ID C_2841533_10, respectively (Applied Biosystems, Nærum, Denmark).

*SELENOP*/rs7579: primers: F: 5′-CAA AAA AGT GAG AAT GAC CTT CAA ACT-3′, R: 5′-ATG CTG GAA ATG AAA TTG TGT CTA GA-3′ (TAGCopenhagen, Copenhagen, Denmark), G-allele: 5′-FAM-AAA ATA G**G**A CAT ACT CCC C-MGB-3′, A-allele: 5′-VIC-AAA TAG **A**AC ATA CTC CCC AAT T-MGB-3′ (Applied Biosystems, Nærum, Denmark).

All samples were determined as duplicates with known positive controls (three for each genotype) and three negative controls containing Milli Q water. All duplicates yielded 100% identical genotypes.

### Statistical methods

The statistical analysis was based on all available observations. Participants who were randomized into a group, but did not attend the baseline appointment (week 0), were excluded. Baseline characteristics are presented as either number with its percentage and medians with 5th and 95th percentiles for each study group. For the polymorphisms, minor allele frequencies are also presented. Outlying observations were identified from visual inspection of the data (correlation plots of week 0 vs. week 13 and week 0 vs. week 26). Deviation from Hardy–Weinberg equilibrium was assessed using a chi-square test.

Linear multiple regression analysis was applied to evaluate the intervention effect on erythrocyte GPX enzyme activity. Within the two groups, mean changes (weeks 0–13, weeks 0–26) and the difference between the groups’ mean changes (weeks 0–26) were calculated from the linear multiple regression. All values of GPX enzyme activity were log-transformed to correct for right-skewed distribution.

To examine the association between mean concentrations of whole blood selenium, plasma selenoprotein P or erythrocyte GPX enzyme activity according to genotype at baseline, week 13 and week 26, respectively, a linear multiple regression analysis was applied using least square means adjusted for baseline concentrations of whole blood selenium, plasma selenoprotein P, or erythrocyte GPX enzyme activity, respectively. Adjustment for baseline level was done to eliminate baseline levels’ influence on the effect of the SNPs. In order to increase the statistical power, heterozygote variant allele and homozygote variant allele carriers were pooled in the analyses.

Moreover, we examined whether sex, BMI, and age modified the relationship between intervention outcomes and the polymorphisms. An interaction term between sex, BMI, and age, respectively, and the studied polymorphisms was therefore included in the model.

A mixed-model, repeated-measures analysis of variance (ANOVA) was used to determine the within-subject effect between genotype and erythrocyte GPX enzyme activity, whole blood selenium, or plasma selenoprotein P concentrations during the entire intervention period.

The statistical analyses were carried out using SAS (release 9.4, SAS Institute, Inc., Cary, NC, USA) and the procedure general linear model (GLM).

For all tests, a *P* value less than 0.05 was considered statistically significant.

## Results

We evaluated the effect of four functional polymorphisms in *GPX1*, *GPX4*, and *SELENOP* on erythrocyte GPX enzyme activity, whole blood selenium, and plasma selenoprotein P concentrations after consumption of 1000 g fish and mussels per week for 26 consecutive weeks (~ 50 μg selenium/day) in volunteer participants [28]. Furthermore, we evaluated the effect of the intervention on erythrocyte GPX enzyme activity.

Baseline characteristics of all participants including the control group are presented in Table 1 as published previously [28], except for GPX enzyme activity measurements, which have not been published previously. None of the baseline variables differed between the two groups (Table 1) or according to genotype (results not shown). The genotype distributions of the studied polymorphisms were in Hardy–Weinberg equilibrium (results not shown).

**Table 1:** Baseline characteristics of the participants presented as either number (%) or median value (5th–95th percentiles)

Variable	MAF (%)	Intervention group (*n* = 49)	Control group (*n* = 45)
Women		21 (43%)	19 (42%)
Men		28 (57%)	26 (58%)
Age, years		61 (51–72)	59 (51–73)
BMI, kg/m^2^		26.3 (21.0–31.1)	25.2 (20.3–32.5)
Whole blood selenium, ng/mL		113.5 (91.3–147.4)	114.6 (96.6–136.0)^1^
Plasma selenoprotein P, ng selenium/mL		51.4 (35.0–63.9)^2^	51.4 (35.4–65.7)^3^
Plasma selenium, ng/mL		84.7 (67.8–106.5)^2^	86.4 (70.5–103.3)^3^
Erythrocyte GPX enzyme activity, U/g Hb		86 (57–153)^4^	84 (54–156)^5^
*GPX1*/rs1050450	29.8		
CC		23 (47%)	22 (49%)
CT		22 (45%)	20 (44%)
TT		4 (8%)	3 (7%)
*GPX4*/rs713041	37.8		
CC		16 (33%)	17 (38%)
CT		25 (51%)	26 (58%)
TT		8 (16%)	2 (4%)
*SELENOP*/rs3877899	19.1		
CC		30 (61%)	30 (67%)
CT		18 (37%)	14 (31%)
TT		1 (2%)	1 (2%)
*SELENOP*/rs7579	30.3		
GG		24 (49%)	19 (42%)
GA		22 (45%)	23 (51%)
AA		3 (6%)	3 (7%)

Part of this table has been published in [28]. MAF, minor allele frequency for the studied population (*n* = 94). ^1^*n* = 44 due to exclusion of outlying values (*n* = 1), all whole blood and plasma selenium and selenoprotein P concentrations for this person were excluded; ^2^*n* = 46 due to errors in the laboratory measures (*n* = 3), all plasma selenoprotein P concentrations and the plasma selenium concentration analyzed only at baseline were excluded for these persons; ^3^*n* = 42 due to errors in the laboratory measures (*n* = 2), all plasma selenoprotein P concentrations and the plasma selenium concentration analyzed only at baseline were excluded for these persons, and due to exclusion of outlying values (*n* = 1). ^4^*n* = 44 since GPX activity was not analyzed on persons having only baseline levels measured (*n* = 1). ^5^*n* = 41 since GPX activity was not analyzed on persons having only baseline levels measured (*n* = 8)

In Table 2, changes in erythrocyte GPX enzyme activity measurements within groups (intervention and control) and differences between group changes are illustrated. There were no statistically significant differences in erythrocyte GPX enzyme activity changes between the intervention and control groups after, neither 13 nor 26 weeks (Table 2).

**Table 2:** Changes within groups and differences between group changes in erythrocyte GPX enzyme activities

	Mean change within group, log (U/g Hb) (95% CI)^a^				Difference between group mean change, log (U/g Hb) (95% CI)*	
	*N*	Weeks 0–13	*n*	Weeks 0–26	*N*	Weeks 0–26
Intervention	41	0.0332 (0.0160, 0.0503)	41	0.0461 (0.0239, 0.0684)	85	0.0263 (− 0.00498, 0.0577)
Control	44	0.0169 (0.000278, 0.0334)	44	0.0198 (− 0.00221, 0.0418)		

^a^Adjusted for baseline levels of GPX enzyme activity

In Table 3, the associations between mean erythrocyte GPX enzyme activity, concentrations of whole blood selenium and plasma selenoprotein P, and the studied polymorphisms, and within-subject effects between genotype and time for the intervention group, are shown. A difference in mean GPX enzyme activity at baseline was found for the *GPX1*/rs1050450 polymorphism among participants who were randomized to the intervention (*P* = 0.044). This difference in enzyme activity persisted after 13 and 26 weeks’ intervention, resulting in no statistically significant difference in response to the increased selenium intake between genotypes. Mean GPX enzyme activity at baseline among CC homozygotes was 104.1 and 84.0 U/g Hb among variant T-allele carriers (intervention group only—results not shown). There was no interaction between any of the studied polymorphisms and whole blood selenium or plasma selenoprotein P concentrations (Table 3). However, there was a statistically significant difference of − 4.68 ng/mL (95% CI − 8.49, − 0.871) between mean concentration of plasma selenoprotein P at week 26 for variant T-allele and CC homozygotes of the *SELENOP*/rs3877899 polymorphism (Table 3). A mean difference in whole blood selenium for the *SELENOP*/rs3877899 polymorphism was also seen; however, this association was not statistically significant (difference between means − 5.76 (95% CI − 12.5, 1.01). Results for the control group are presented in Additional file 2. None of the polymorphisms among participants in the control group differed for either of the outcome measures as expected.

**Table 3:** Association between mean concentrations of erythrocyte GPX enzyme activity, whole blood selenium, and selenoprotein P in relation to the studied polymorphisms, and within-subject effects between genotype and time in the intervention group

Erythrocyte GPX enzyme activity, log (U/g Hb)								
SNP	*n*	Baseline		13 weeks^c^		26 weeks^c^		*P* value for interaction between genotype and time
		Difference between means (95% CI)	*P* value	Difference between means (95% CI)	*P* value	Difference between means (95% CI)	*P* value	
*GPX1*/rs1050450								
CC	20	− 0.224 (− 0.442, − 0.00638)	0.044	0.0299 (− 0.00981, 0.0696)	0.14	− 0.00585 (− 0.06278, 0.0511)	0.84	0.085
CT + TT	21							
*GPX4*/rs713041								
CC	15	0.129 (− 0.105, 0.364)	0.27	− 0.00532 (− 0.0462, 0.0355)	0.79	− 0.0143 (− 0.0711, 0.0424)	0.61	0.79
CT + TT	26							
*SELENOP*/rs3877899								
CC	25	− 0.0228 (− 0.258, 0.212)	0.85	− 0.00617 (− 0.0459, 0.0335)	0.75	0.0145 (− 0.0407, 0.0697)	0.60	0.70
CT + TT	16							
*SELENOP*/rs7579								
GG	22	− 0.0278 (− 0.258, 0.202)	0.81	− 0.0134 (− 0.0521, 0.0252)	0.49	− 0.0143 (− 0.0683, 0.0397)	0.60	0.81
GA + AA	19							
Whole blood selenium, ng/mL								
SNP	n*	Baseline		13 weeks^d^		26 weeks^d^		*P* value for interaction between genotype and time
		Difference between means (95% CI)	*P* value	Difference between means (95% CI)	*P* value	Difference between means (95% CI)	*P* value	
*GPX1*/rs1050450								
CC	23	3.20 (− 5.46, 11.9)	0.46	− 0.989 (−7.89, 5.91)	0.77	− 1.24 (− 8.11, 5.63)	0.72	0.83
CT + TT	26							
*GPX4*/rs713041								
CC	16	4.85 (− 4.32, 14.0)	0.29	− 2.76 (− 9.91, 4.38)	0.44	− 1.77 (− 8.93, 5.39)	0.62	0.54
CT + TT	33							
*SELENOP*/rs3877899								
CC	30	0.989 (− 7.93, 9.91)	0.82	0.675 (− 6.37, 7.72)	0.85	− 5.76 (− 12.5, 1.01)	0.093	0.13
CT + TT	19							
*SELENOP*/rs7579								
GG	24	4.44 (− 4.16, 13.0)	0.30	− 4.04 (− 10.9, 2.80)	0.24	1.60 (− 5.32, 8.53)	0.64	0.23
GA + AA	25							
Selenoprotein P, ng/mL								
SNP	*n* ^*b*^	Baseline		13 weeks^e^		26 weeks^e^		*P* value for interaction between genotype and time
		Difference between means (95% CI)	*P* value	Difference between means (95% CI)	*P* value	Difference between means (95% CI)	*P* value	
*GPX1*/rs1050450								
CC	21	4.17 (− 0.51, 8.85)	0.080	− 1.17 (− 5.38, 3.04)	0.58	− 2.56 (− 6.60, 1.47)	0.21	0.071
CT + TT	25							
*GPX4*/rs713041								
CC	16	3.49 (− 1.47, 8.45)	0.16	0.801 (− 3.41, 5.01)	0.70	− 1.17 (− 5.32, 2.97)	0.57	0.48
CT + TT	30							
*SELENOP*/rs3877899								
CC	28	− 2.33 (− 7.23, 2.57)	0.34	− 2.43 (− 6.53, 1.67)	0.24	− 4.68 (− 8.49, − 0.871)	0.018	0.33
CT + TT	18							
*SELENOP*/rs7579								
GG	23	2.16 (− 2.63, 6.94)	0.37	1.72 (− 2.35, 5.79)	0.40	− 0.630 (− 4.66, 3.40)	0.75	0.79
GA + AA	23							

^a^13 and 26 weeks’ measurements only included 41 participants due to discontinuation of intervention

^b^13 and 26 weeks’ measurements only included 38 participants due to discontinuation of intervention

^c^Adjusted for baseline levels of erythrocyte GPX enzyme activity

^d^Adjusted for baseline levels of whole blood selenium

^e^Adjusted for baseline levels of selenoprotein P

**Fig. 1:**
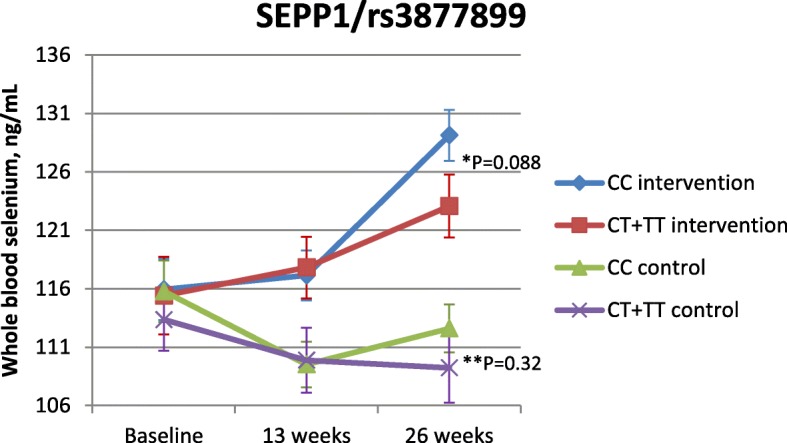
Association between mean concentrations of whole blood selenium and the *SELENOP*/rs3877899 polymorphism. Mean concentrations of whole blood selenium ± SD for the intervention and control group estimated by linear multiple regression adjusted for baseline level of whole blood selenium. *P* values for difference in mean whole blood selenium concentrations at week 26 for genotype effect within the intervention group (*) and within the control group (**), respectively, are illustrated

**Fig. 2:**
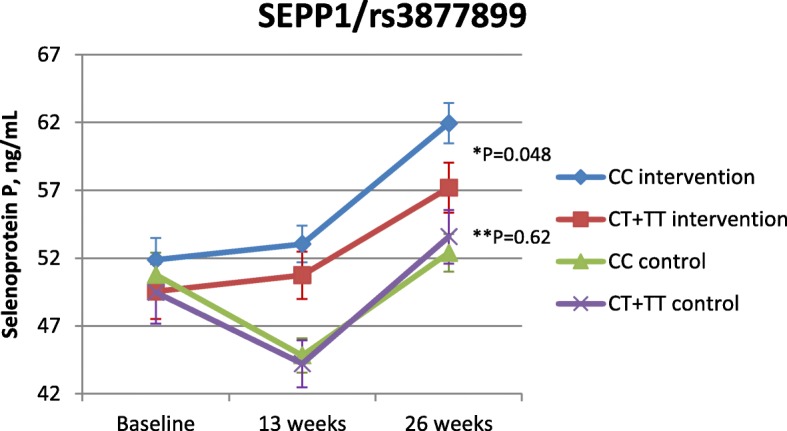
Association between mean concentrations of selenoprotein P and the *SELENOP/*rs3877899 polymorphism. Mean concentrations of selenoprotein P ± SD for the intervention and control group estimated by linear multiple regression adjusted for baseline level of selenoprotein P. *P* values for difference in mean selenoprotein P concentrations at week 26 for genotype effect within the intervention group (*) and within the control group (**), respectively, are illustrated

In order to elucidate whether these differences in selenoprotein P and whole blood selenium concentrations were due to chance or an effect of the intervention, we examined the association between the *SELENOP*/rs3877899 polymorphism and whole blood selenium and selenoprotein P concentrations, respectively, with the control group included in the model (Figs. 1 and 2). After 26 weeks, the difference in whole blood selenium mean concentrations between CC homozygotes and T-allele carriers was only borderline statistically significant (*P* = 0.088) (Fig. 1), whereas the mean difference in selenoprotein P concentrations between CC homozygotes and T-allele carriers of the *SELENOP*/rs3877899 polymorphism was still statistically significant (*P* = 0.048) (Fig. 2).

We also tested whether sex, age, or BMI modified the effect on the intervention for the studied polymorphisms, but we did not find any sign of such effect modification (results not shown).

## Discussion

The present study showed that CC homozygotes of the *SELENOP*/rs3877899 polymorphism who consumed 1000 g fish and mussels per week for 26 consecutive weeks had higher levels of both selenoprotein P and whole blood selenium compared to fish and mussel consuming variant T-allele carriers and the control group although the effect in whole blood selenium concentration was not statistically significant.

To our knowledge, this is the first study investigating the effect of *SELENOP* and *GPX* polymorphisms after ingestion of high selenium content foods in a controlled randomized trial. The SELGEN study [19] examined the effect on plasma selenium and selenoprotein P concentrations of the same *SELENOP* polymorphisms as in the present study before and after selenium supplementation in a UK population also known to be low in selenium status. Mean baseline plasma selenium concentrations in the two studies were comparable (1.15 μmol/l corresponding to 90.8 ng/mL in the SELGEN study and 85.8 ng/mL in the present study). The two investigated *SELENOP* polymorphisms significantly affected plasma selenium concentration after 6 weeks of supplementation with 100 μg selenium/day as sodium selenite [19]. The authors noted that the effect was strongest among participants with a BMI exceeding 30 where *SELENOP*/rs3877899 CC homozygotes (referred to as GG in [19]) and variant A-allele carriers of *SELENOP*/rs7579 responded better to selenium supplementation compared to carriers of the variant T-allele (referred to as A-allele in [19]) and GG homozygotes, respectively. The same study reported a statistically significant increase in plasma selenoprotein P concentration following selenium supplementation and gender-specific differences in baseline concentrations and post-supplementation with *SELENOP*/rs3877899 and *SELENOP*/rs7579, respectively. Lower baseline concentration of plasma selenoprotein P was measured in women who were heterozygous T-allele carriers of the *SELENOP*/rs3877899 polymorphism compared to men with corresponding genotype, while the opposite applied for CC homozygotes. Among carriers of the *SELENOP*/rs7579 variant A-allele, men had higher plasma selenoprotein P concentrations post-supplementation compared to women. We were not able to reproduce the findings on gender and BMI which could be due to a lack of power to study gene-environment interactions in the present study given the relatively small sample size. Our study differs from the SELGEN intervention in several ways which might contribute to the different findings. Besides a longer study period (26 weeks compared to 6 weeks in the SELGEN study), different sources of selenium and dosage levels were used in the interventions. We supplemented with a dietary source of selenium, while selenite was used in the SELGEN study, and at a dosage level that was half the one given by Méplan et al. [19]. Compared to inorganic selenium, organic selenium compounds, such as selenomethionine present in fish and mussels, are non-specifically incorporated into the general protein pool, leaving a smaller part of the absorbed selenium readily available for biosynthesis of selenoproteins. However, it is not clear whether this had an impact on the results, given the long supplementation period and the relatively high bioavailability of selenium from fish which has been reported to be between 56 and 90% [11, 12, 38].

Increasing the intake of selenium with fish and mussels did not affect erythrocyte GPX enzyme activity, suggesting that this selenoprotein was maximally expressed prior to supplementation. This is consistent with previous results, showing that the antioxidant enzyme GPX is saturated at blood selenium concentrations around 100 ng/mL [27, 46]. When stratifying the data according to genotype, in line with previous findings [34], carriers of the variant *GPX1* allele had significantly lower erythrocyte GPX activity compared to CC homocygotes. A compromised antioxidant defense may lead to increased susceptibility to oxidative stress and DNA damage [25].

The increase in selenoprotein P concentration in the control group from week 13 to week 26 is unexpected and is difficult to explain (Fig. 2). This has been discussed in more detail in the main paper that evaluated the intervention effect on blood selenium status [28].

Strengths and limitations of the overall study design were thoroughly described by Outzen et al. [28]. Shortly, the strengths of the study are the design with the randomization procedure, which ensure evenly distribution of confounders, the restrictive inclusion and exclusion criteria, and the long-term duration of the intervention combined with the high compliance. Limitations include lack of blinding and that participants were non-fasting at blood sampling. However, we based our results on whole blood which has been shown to reflect the long-term selenium status [1, 17] as opposed to plasma selenium, which has a short half-life [5]. With regard to the lack of blinding, this is not expected to influence on this study since we mainly studied the effect of genetic variation in the intervention group. We are well aware of the present study being underpowered due to the small sample size. Nevertheless, our study is in accordance with the SELGEN study, and we are therefore able to support the notion that variation in the *SELENOP* gene affects selenium biomarker concentration after intake of both a selenium dietary supplement and food with a high content of selenium.

## Conclusion

Taken together, our study indicates that genetically determined variation in *SELENOP* leads to different responses in expression of selenoproteins following consumption of selenium-rich foods. This emphasizes the importance of taking individual aspects such as genotypes into consideration when assessing risk in public health recommendations, since they may affect selenium metabolism and response to selenium intake and thereby enable a more personalized approach to micronutrient requirements.

## Additional files

Additional file 1: Flow chart of study participants as previously published [28]. (DOCX 37 kb)

Additional file 2: Association between mean concentrations of erythrocyte GPX enzyme activity, whole blood selenium and selenoprotein P in relation to the studied polymorphisms, and within-subject effects between genotype and time in the control group. (DOCX 24 kb)

## Abbreviations

BMI: Body mass index
GPX: Glutathione peroxidases
SNP: Single nucleotide polymorphism
