# Supplementary material for: Genetic polymorphism in selenoprotein P modifies the response to selenium-rich foods on blood levels of selenium and selenoprotein P in a randomized dietary intervention study in Danes
Source: Genes Nutr. 2018 Jul 13;13:20. doi: 10.1186/s12263-018-0608-4 (PMC6045871; doi:10.1186/s12263-018-0608-4)
Supplement: Supplementary file 1 — Flow chart of study participants as previously published [28]. (DOCX 37 kb) [file 12263_2018_608_MOESM1_ESM.docx]

Week 13 (n=44)

Week 0 (n=45)

Assessed for eligibility

from a questionnaire (n=115)

Returned a signed declaration
of consent (n=102)

Assigned to
control group

(n=51)

Assigned to intervention group (n=51)

Week 0 (n=51)

Week 26 (n=42)

Week 13 (n=43)

Week 26 (n=42)

Randomized (n=102)

Did not attend appointment (n=6)

Discontinued intervention (n=8)

- breast cancer
 diagnosis (n=1)

- wife discontinued

participation (n=1)

- dropped out (n=2)

- discomfort with the

intervention (n=2)

- colorectal cancer
 diagnosis (n=1)

- use of heart disease

medication (n=1)

Use of diabetic medication (n=1)

Did not attend appointment (n=2)

Use of heart disease medication (n=1)

(n=1)

Completed (n=42)

Completed (n=41)

Intake of selenium supplement (n=1)
