# Supplementary material for: Genetic polymorphism in selenoprotein P modifies the response to selenium-rich foods on blood levels of selenium and selenoprotein P in a randomized dietary intervention study in Danes
Source: Genes Nutr. 2018 Jul 13;13:20. doi: 10.1186/s12263-018-0608-4 (PMC6045871; doi:10.1186/s12263-018-0608-4)
Supplement: Supplementary file 2 — Association between mean concentrations of erythrocyte GPX enzyme activity, whole blood selenium and selenoprotein P in relation to the studied polymorphisms, and within-subject effects between genotype and time in the control group. (DOCX 24 kb) [file 12263_2018_608_MOESM2_ESM.docx]

**Additional File 2:** Association between mean concentrations of erythrocyte GPX enzyme activity, whole blood selenium and selenoprotein P in relation to the studied polymorphisms, and within-subject effects between genotype and time in the control group.

| Erythrocyte GPX enzyme activity, log (U/g Hb) | | | | | | | | |
| --- | --- | --- | --- | --- | --- | --- | --- | --- |
| *SNP* | *n* | *Baseline* | | *13 weeks**** | | *26 weeks**** | | *P-value for interaction between genotype and time* |
|  |  | *Difference between means (95% CI)* | *P-value* | *Difference between means (95% CI)* | *P-value* | *Difference between means*  *(95% CI)* | *P-value* |  |
| *GPX1*/rs1050450  CC  CT+TT | 21  23 | -0.0649 (-0.124;-0.253) | 0.49 | 0.00206 (-0.0279;0.0320) | 0.89 | -0.0159 (-0.0187;0.0505) | 0.36 | 0.80 |
| *GPX4*/rs713041  CC  CT+TT | 16  28 | -0.167 (-0.356;0.0233) | 0.084 | 0.00391 (-0.0282;0.0356) | 0.81 | 0.00107 (-0.0366;0.0388) | 0.95 | 0.63 |
| *SELENOP*/rs3877899  CC  CT+TT | 29  15 | -0.1176 (-0.314;0.0787) | 0.23 | -0.0142 (-0.0458;0.0175) | 0.37 | 0.00198 (-0.0355;0.0394) | 0.92 | 0.77 |
| *SELENOP*/rs7579  GG  GA+AA | 19  25 | -0.0718 (-0.262;0.118) | 0.45 | 0.0179 (-0.0118;0.0477) | 0.23 | 0.0225 (-0.0121;0.0571) | 0.20 | 0.24 |
| Whole blood selenium, ng/mL | | | | | | | | |
| *SNP* | *n** | *Baseline* | | *13 weeks***** | | *26 weeks***** | | *P-value for interaction between genotype and time* |
|  |  | *Difference between means*  *(95% CI)* | *P-value* | *Difference between means (95% CI)* | *P-value* | *Difference between means (95% CI)* | *P-value* |  |
| *GPX1*/rs1050450  CC  CT+TT | 21  23 | -2.36 (-10.1;5.33) | 0.54 | -0.401 (-6.16;5.36) | 0.89 | 4.03 (-2.41;10.5) | 0.21 | 0.15 |
| *GPX4*/rs713041  CC  CT+TT | 16  28 | -4.91 (-12.8;2.97) | 0.22 | -2.66 (-8.68;3.36) | 0.38 | 0.113 (-6.97;7.20) | 0.97 | 0.99 |
| *SELENOP*/rs3877899  CC  CT+TT | 29  15 | -0.979 (-9.12;7.16) | 0.81 | 0.820 (-5.20;6.83) | 0.78 | -3.78 (-10.6;3.04) | 0.27 | 0.48 |
| *SELENOP*/rs7579  GG  GA+AA | 18  26 | 1.48 (-6.36;9.32) | 0.71 | -0.741 (-6.55;5.07) | 0.80 | 2.04 (-4.59;8.67) | 0.54 | 0.80 |
| Selenoprotein P, ng/mL | | | | | | | | |
| *SNP* | *n*** | *Baseline* | | *13 weeks****** | | *26 weeks****** | | *P-value for interaction between genotype and time* |
|  |  | *Difference between means (95% CI)* | *P-value* | *Difference between means (95% CI)* | *P-value* | *Difference between means (95% CI)* | *P-value* |  |
| *GPX1*/rs1050450  CC  CT+TT | 19  23 | -3.08 (-8.78;2.63) | 0.28 | -0.739 (-5.24;3.76) | 0.74 | 0.299 (-5.26;5.85) | 0.91 | 0.83 |
| *GPX4*/rs713041  CC  CT+TT | 16  26 | 2.75 (-8.62;3.12) | 0.35 | 0.156 (-4.47;4.78) | 0.95 | -2.46 (-8.09;3.17) | 0.38 | 0.72 |
| *SELENOP*/rs3877899  CC  CT+TT | 28  14 | -1.31 (-7.40;4.79) | 0.67 | -0.578 (-5.26;4.10) | 0.80 | 1.14 (-4.66;6.93) | 0.69 | 0.80 |
| *SELENOP*/rs7579  GG  GA+AA | 17  25 | 0.457 (-6.32;5.41) | 0.88 | 3.26 (-1.13;7.65) | 0.14 | 4.82 (-0.485;10.1) | 0.074 | 0.19 |

* 13 and 26 weeks measurements only included 44 participants due to discontinuation of intervention

** 13 and 26 weeks measurements only included 42 participants due to discontinuation of intervention

***Adjusted for baseline levels of erythrocyte GPX enzyme activity

****Adjusted for baseline levels of whole blood selenium

*****Adjusted for baseline levels of selenoprotein P
